# Deterministic radiative coupling of two semiconductor quantum dots to the optical mode of a photonic crystal nanocavity

**DOI:** 10.1038/s41598-017-03989-y

**Published:** 2017-06-22

**Authors:** M. Calic, C. Jarlov, P. Gallo, B. Dwir, A. Rudra, E. Kapon

**Affiliations:** Laboratory of Physics of Nanostructures, Ecole Polytechnique Fédérale de Lausanne (EPFL), CH-1015 Lausanne, Switzerland

## Abstract

A system of two site-controlled semiconductor quantum dots (QDs) is deterministically integrated with a photonic crystal membrane nano-cavity. The two QDs are identified via their reproducible emission spectral features, and their coupling to the fundamental cavity mode is established by emission co-polarization and cavity feeding features. A theoretical model accounting for phonon interaction and pure dephasing reproduces the observed results and permits extraction of the light-matter coupling constant for this system. The demonstrated approach offers a platform for scaling up the integration of QD systems and nano-photonic elements for integrated quantum photonics applications.

## Introduction

Studies of the radiative coupling of single semiconductor quantum dots (QDs) to photonic cavities unravel cavity quantum electrodynamic (c-QED) effects in the solid state^1, 2^ and suggest robust and scalable platforms for integrated quantum photonics, e.g. for quantum information technologies^3–5^. Scaling up these investigations to multi-QD systems is essential for implementing promising architectures based on quantum networks^6, 7^ or multiplexed single photon sources^8^. Although first steps in this direction have been taken using self-assembled QDs, demonstrating weak or strong coupling of pairs of dots to a mode of a micropillar^9–11^, microdisk^12^ or photonic crystal (PhC) cavity^13–17^, systematic scaling requires technologies capable of deterministic QD-cavity integration^18–20^.

In this work, we demonstrate such deterministic radiative coupling of two site-controlled QDs a PhC cavity, manifested by the observation of co-polarization and Purcell enhancement of their photoluminescence and confirmed with a microscopic model^21^. These results illustrate a scalable approach to QD-based integrated quantum photonics.

## Results

**Figure 1:**
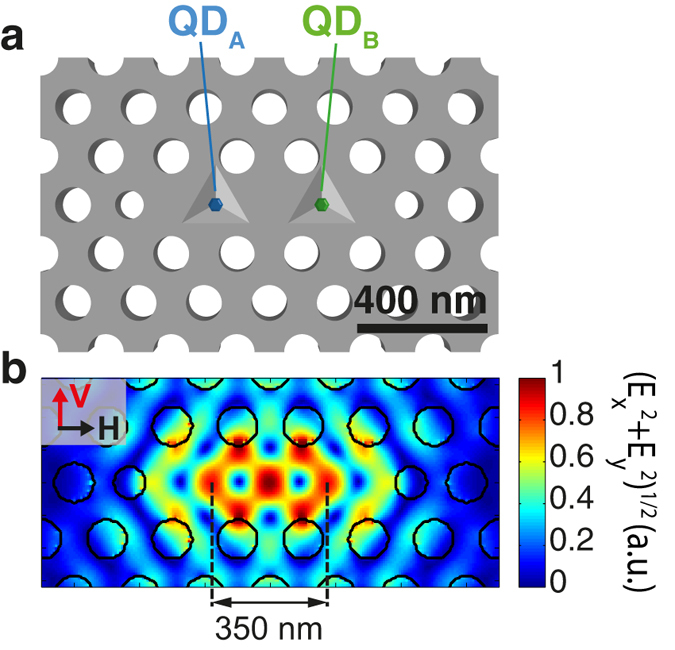
Deterministic integration of two pyramidal QDs with a modified L_3_ PhC membrane cavity. (**a**) Schematic illustration. (**b**) Simulated near field intensity pattern of the fundamental optical mode. The distance between the two QDs (350 nm) is equal to that of the field lobes as indicated.

**Figure 2:**
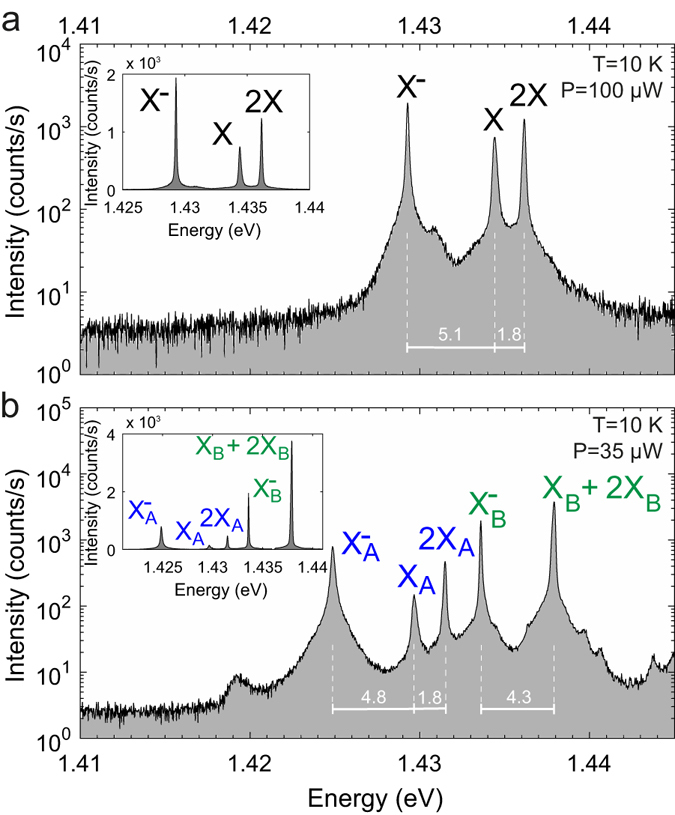
Comparison of PL semilogarithmic spectra of pyramidal QDs in off-resonance PhC L_3_ cavities. (**a**) Typical off-resonant spectrum of a single QD at low excitation. (**b**) Two QDs with corresponding spectral lines labeled A and B. The neutral exciton *X*_*B*_ and the bi-exciton 2*X*_*B*_ lines overlap in the case of *QD*_*B*_. Separations of spectral features are indicated in *meV*. Insets: Corresponding linear scale spectra.

**Figure 3:**
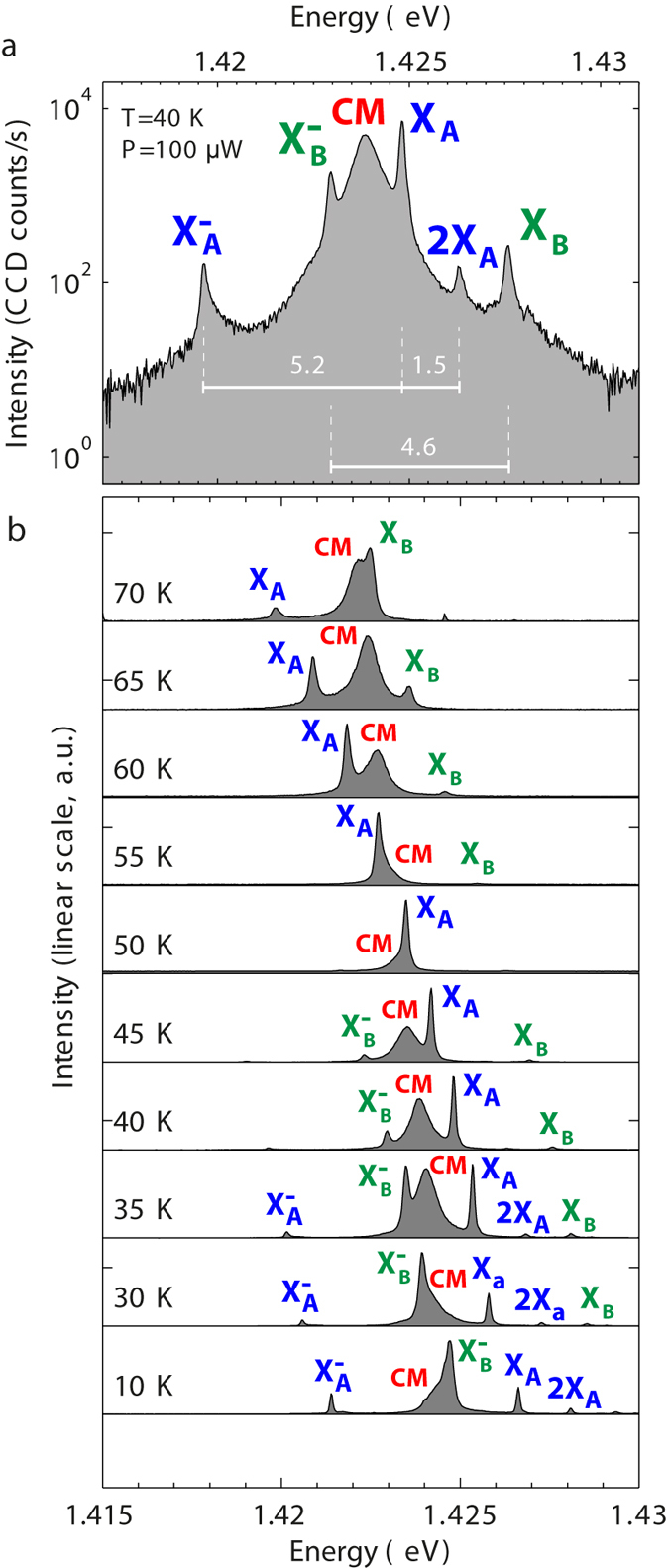
PL spectra of two QDs coupled to the same PhC cavity mode. (**a**) Semi-logarithmic plot showing the cavity mode CM tuned spectrally in between the exciton lines belonging to two spatially separated QDs A and B. Energy separations are indicated in meV. (**b**) Temperature dependence (10–70 *K*) of the PL spectra (normalized to maximum intensity).

The double-QD structure integrated with a modified L_3_ PhC membrane cavity (Fig. 1) was produced using site-controlled InGaAs/GaAs pyramidal QD structures, which ensured a mutual alignment accuracy of ~50 *nm* ^21–23^. In the vicinity of the double-QD structures, additional control structures containing a single QD at the center of an L_3_ PhC cavity were implemented (same design as in Fig. 1b). At sufficiently low excitation power, the typical off-resonance micro-photoluminescence (*μ*PL) spectrum of such a control structure appears as shown in Fig. 2a. This spectrum features only 3 peaks: the neutral exciton (*X*), the negatively charged exciton (*X* ^−^) and the biexciton (2*X*). This spectral “fingerprint” of a single QD is highly reproducible in terms of the energy separation between the *X* and the *X* ^−^ lines, statistically evaluated to be 4.9 ± 0.3 *meV* on this specific sample^24^. Indeed, the *μ*PL spectrum of the double-QD system seen in Fig. 2b, also far detuned from the cavity mode (CM) resonance, exhibits spectral lines in which the *X* and *X* ^−^ of each dot can easily be identified by comparison with Fig. 2a. The binding energy of the 2X, on the other hand, is more sensitive to the details of the QD heterostructure, varying between +1 *meV* to −4 *meV*, which is also reflected in the double-QD spectra^24^. The energy difference between the neutral excitons of dots A and B is consistent with the inhomogeneous broadening of the QD ensemble (i.e. the spectral width of the QD ensemble emission, which is ~10 *meV*, including *X* and *X* ^−^ populations) as determined from *μ*PL spectra of QD ensembles. Contrary to previous studies based on self-assembled QDs lacking site control^9–11, 13–17^, our deterministic integration yields unambiguous evidence for the optical coupling of the QD pair with the CM. Figure 3 shows the spectrum of two pyramidal QDs (A and B) tuned in resonance with the fundamental mode (CM) of the L_3_ cavity by varying the sample temperature. All spectral features of the two QD excitons and the cavity mode (*Q* = 2200), are unequivocally identified. At 40 *K*, the neutral exciton *X*_*A*_ of dot A and the negatively charged exciton ${X}_{B}^{-}$ of dot B simultanously overlap with the CM.

**Figure 4:**
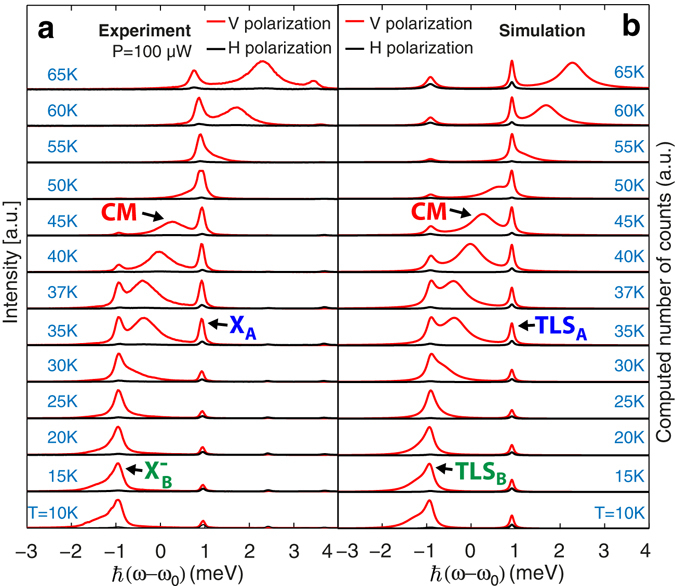
PL spectra of two QDs coupled to the same CM. Comparison of measured (**a**) and calculated (**b**) spectra of the coupled 2QDs-cavity system at different sample temperatures, resolved in linear polarization (see Fig. 1d for definition of V and H polarizations). Spectra are normalized by the maximum intensity and ${\omega }_{0}=({\omega }_{{X}_{B}^{-}}+{\omega }_{{X}_{A}})\mathrm{/2}$. The two-level system (TLS) component is indicated.

Evidence for (weak) coupling of both QD excitons to the common CM is presented in Fig. 4a,b, which depicts the measured and modeled (see Supplementary material part A) polarization-resolved PL spectra of the structure, where *I*_*V*_ and *I*_*H*_ are the spectra resolved in linear polarization along the vertical (V) and horizontal (H) directions indicated in Fig. 1b. The measured spectra focus on the ${X}_{B}^{-}$ and *X*_*A*_ features, each spectrum centered at the average frequency of these two transitions ${\omega }_{0}=({\omega }_{{X}_{B}^{-}}+{\omega }_{{X}_{A}})\mathrm{/2}$. The CM is resonant with ${X}_{B}^{-}$ at *T* ~ 20 *K* and with *X*_*A*_ at *T*~ 55 *K*. With decreasing detuning, the QD excitons increasingly acquire the V polarization of the CM, as for single-QD cavity structures^22, 25^. The very good agreement with the calculated spectra substantiates that both QDs are radiatively coupled to the same CM. The fixed simulation parameters extracted from the experimental data are the QD spontaneous emission rates $\hslash {\gamma }_{A}=\hslash {\gamma }_{B}=0.2\,\mu eV$, the QD pure dephasing rates $\hslash {\gamma }_{A}^{d}=104\,\mu eV$ and $\hslash {\gamma }_{B}^{d}=230\,\mu eV$, the cavity mode loss rate $\hslash \kappa =650\,\mu eV$, and the fitting parameters are the incoherent QD pumping rate $\hslash P=1\,\mu eV$, the QD-cavity coupling strengths $\hslash {g}_{0}^{A}=\hslash {g}_{0}^{B}=50\,\mu eV$ and the phonon scattering rate amplitude $2\pi A/{\hslash }^{2}=1.4\,nm/meV$ (see Supplementary material part A). The CM off-resonant emission, which results from pure dephasing and phonon scattering^26, 27^, the enhanced QD emission intensity near resonance (see Supplementary material Fig. S1 for details) and the QD lines co-polarization behavior are accounted for by the simulations.

**Figure 5:**
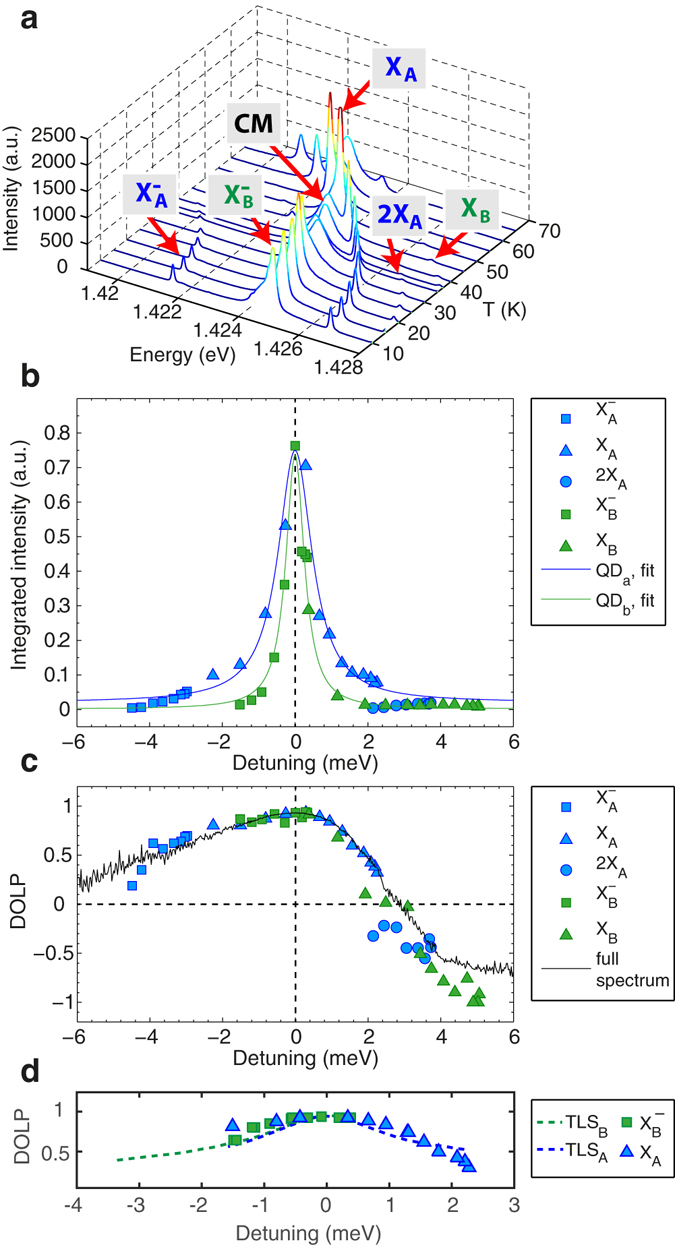
PL intensity and DOLP detuning dependence. (**a**) PL spectra (not normalized) of the 2QDs-cavity structure versus temperature. Integrated intensities (**b**) and degree of linear polarization (DOLP) (**c**) of the excitonic emission peaks versus detuning with respect to the cavity mode. In (**b**), the blue (green) solid lines represent Lorentzian fits to the data points of the *X* and *X* ^−^ features of *QD*_*A*_ (*QD*_*B*_). In (**c**), the solid black line displays the DOLP of the full spectrum, acquired at 40 *K* high above saturation (*P* = 5 *mW*). (**d**) Comparison of the measured DOLP of the ${X}_{B}^{-}$ and *X*_*A*_ transitions with simulated values. In (**b**–**d**), the detuning is defined as $\hslash {\delta }_{j}=\hslash {\omega }_{j}-\hslash {\omega }_{cav}$.

The variation in the intensities of the different spectral features near resonance is depicted in Fig. 5a,b, which shows the temperature dependence of the (un-normalized) PL spectra as well as the integrated intensities of each line. The intensities of the peaks belonging to QD A and B versus detuning, defined as $\hslash {\delta }_{j}=\hslash {\omega }_{j}-\hslash {\omega }_{cav}$, trace nearly perfect Lorentzians with FWHM of ~1 *meV* and ~0.6 *meV*, respectively. This behavior is well explained by the dependence of the Purcell factor on detuning, which can be approximated by a Lorentzian function

1 $${F}_{P} \sim \frac{1+2Q{\gamma }_{j}^{d}/{\omega }_{j}}{8{\delta }^{2}/{\kappa }^{2}+2{(1+2Q{\gamma }_{j}^{d}/{\omega }_{j})}^{2}}$$

for a QD transition broadened by pure dephasing^28^. The only exception to this Lorentzian trend in Fig. 5b is exhibited by the 2*X*_*A*_ line, which can be explained by its conditional dynamics with *X*_*A*_. That is, as the radiative lifetime of the *X*_*A*_ is shortened via the Purcell effect, it leads to a depletion of the 2*X*_*A*_ state and hence its intensity deviates from the Lorentzian dependence versus detuning. Remarkably, the PL intensities of all excitonic species belonging to the same QD are influenced by the cavity in the same way, implying they experience similar coupling strength with the CM.

Figure 5c shows the detuning dependence of the degree of linear polarization $DOLP=({I}_{V}-{I}_{H})/({I}_{V}+{I}_{H})$ for all observed peaks. In this case of a strongly polarized CM and a symmetric QD showing otherwise isotropic polarization of emission in the cavity plane, the QD emission becomes co-polarized with the CM for sufficiently small detuning, a consequence of the Purcell effect^21^. Strikingly, their DOLP follows an s-shaped curve with a maximum value at resonance of ~0.9, corresponding to the DOLP of the CM^29^. This co-polarization behavior of the QD lines with the CM feature at resonance is a consequence of the Purcell effect and is consistent with our observations for single QDs in *L*_3_ PhC cavities^22^. The polarization of individual peaks is in excellent agreement with the DOLP of the full spectrum (solid line) acquired at 40 *K* for high pump powers (*P* = 5 *mW*) such that all excitonic features merge into a quasi-continuum. The simulated DOLP of the TLSs transitions are also in good agreement with the experiment (Fig. 5d). The peculiar shape of the DOLP may be due to a Fano-like interference between the emission of the QD and the CM^30^.

Another special attribute of this coupled 2QDs-cavity system is that the emission intensity of the CM saturates at an excitation power that is about an order of magnitude above that of the excitonic ground states of the two QDs (see Supplemental Fig. SP1). This behavior is disctinct from the phenomenology of single QDs coupled to an L_3_ cavity^22^ and could indicate the onset of cooperative emission. Alternatively, this behavior may be explained as the result of CM feeding from higher energy quantum wire states that are spatially located in the vicinity of pyramidal QDs^28^.

## Conclusion

We demonstrated the deterministic coupling of two site-controlled QDs integrated in an L_3_ PhC cavity. The weak coupling of both QDs to the same CM was revealed through co-polarization and Purcell enhancement of their emission near resonance. These observations were reproduced by a theoretical model that accounts for QD phonon scattering and pure dephasing decoherence mechanisms, from which a QD-cavity coupling strength *g* = 50 *μeV* was extracted for both QDs. The present Letter consolidates the potential of site-controlled QDs as a scalable platform for realizing more advanced cavity QED schemes, such as multiple QDs in a cavity^31^, coupled cavity structures^32^ and waveguide-coupled distant cavities^33^.

## Methods

### Sample Fabrication

The QD-cavity structures were fabricated by first growing arrays of pyramidal InGaAs/GaAs QDs using metallorganic vapor phase epitaxy (MOVPE) on a patterned (111)B GaAs “membrane” wafer^34, 35^. The membrane wafer consisted of a 1 *μm* thick sacrificial AlGaAs layer (Al content ~0.7) overgrown with a 265 *nm* thick GaAs membrane layer, on which triangular arrays of inverted pyramids with 400 *nm* pitch and 300 *nm* side length were fabricated using electron beam lithography (EBL) and wet chemical etching^36^. The grown heterostructure consisted of a 4.3 *nm* thick GaAs buffer layer followed by a 0.2 *nm* thick layer of In_0.2_Ga_0.8_As resulting in the formation of a single symmetric^37, 38^ QD at the apex of each pyramidal pit. The QDs were then capped by growing a 2.5 *nm* thick GaAs layer. All thicknesses correspond to their equivalent on a (100) GaAs planar substrate. The photoluminescence (PL) spectra of the resulting QD ensembles were measured and the emission wavelengths were used in designing PhC modified L_3_ cavities with resonances matching the QD spectra. The PhC cavities were implemented on the grown wafer using PhC hole patterns with period *a* = 200 *nm* and systematic variations in hole radii *r* using EBL and inductively coupled plasma (ICP) dry etching^39^, with the help of mutual alignment marks made on the substrate prior to epitaxial growth. The PhC hole patterns were designed so that all QDs were removed at this step, except for two QDs at the center of the cavity. Scanning electron microscope (SEM) inspection of the resulting structures allows us to estimate positioning accuracy of the dots relative to the cavity pattern of better than 50 *nm*. Note that the separation of the two isolated QDs is 350 *nm*, designed to place each dot at a lobe of the optical near field pattern of the fundamental cavity mode (see Fig. 1b). Such QD separation excludes possible dipole-dipole interactions for inter-dot coupling. The fabrication process ends by membrane release via wet etching in buffered hydrofluoric acid^22, 40^.

### Optical setup

The PL spectra were acquired using a standard *μ*PL setup. The sample was excited using a 730 *nm* wavelength Ti-sapphire laser beam, focused on the sample’s surface to a circular spot with a ~1 *μm* radius using a 50× magnification microscope objective. An excitation power level of *P* = 1 *μW* mentioned in the manuscript corresponds to a power density of 0.5 *W*/*cm* ^2^ applied at the sample’s surface. The PL was then collected using the same microscope objective and dispersed by an 80 *μeV*-resolution spectrometer. A half-wave plate followed by a polarizer was placed in the detection path for the measurements of spectra resolved in linear polarization.

## Electronic supplementary material

Supplementary information
