# Supplementary material for: Deterministic radiative coupling of two semiconductor quantum dots to the optical mode of a photonic crystal nanocavity
Source: Sci Rep. 2017 Jun 22;7:4100. doi: 10.1038/s41598-017-03989-y (PMC5481339; doi:10.1038/s41598-017-03989-y)
Supplement: Supplementary file 1 — Supplementary information [file 41598_2017_3989_MOESM1_ESM.pdf]

Deterministic radiative coupling of two semiconductor quantum dots to the optical mode of a photonic crystal nanocavity

M. Calic, C. Jarlov, P. Gallo, B. Dwir, A. Rudra, and E. Kapon*
Laboratory of Physics of Nanostructures, Ecole Polytechnique Fédérale de Lausanne (EPFL), CH-1015 Lausanne, Switzerland

* eli.kapon@epfl.ch

SUPPLEMENTARY MATERIAL

A. Model of two QDs coupled to a single CM

The coupled system, comprising two QDs and a cavity mode (CM) is modeled by two two-level systems (TLSs) with frequencies $\omega_{j=1,2}/2\pi$ coupled to a single CM at frequency $\omega_{cav}/2\pi$. The TLS-CM energy detuning is $\hbar\delta_j = \hbar\omega_j - \hbar\omega_{cav}$. This approach has been justified by examining the behavior of a single pyramidal QD in an L3 cavity using a similar model[1], demonstrating the TLS nature of these QDs. The TLSs model excitons are assumed to have dipoles oriented at an angle θ with respect to the CM electric field at the QDs positions. This system is described by the Tavis-Cummings Hamiltonian[2]:

$$H^\theta = \hbar\omega_{cav} a^\dagger a + \sum_{j=1,2} \hbar[\omega_j \sigma_+^j \sigma_-^j + \cos\theta g_0^j (a^\dagger \sigma_-^j + a\sigma_+^j)] \tag{1}$$

where σ_-^j (σ_+^j) are the lowering (raising) operators of the two TLSs and a ($a^\dagger$) is the lowering (raising) operator of the CM. The evolution of the density matrix ρ of the coupled system formed by the TLSs and the CM is given by

$$\dot\rho = -\frac{i}{\hbar}[H^\theta, \rho] - \sum_{j=1,2}\Big[L^j(\sqrt{\gamma_j}\sigma_-^j) + L_j(\sqrt{P}\sigma_+^j) + L_j(\sqrt{\gamma_j^d}\sigma_z^j) + L_j(\sqrt{\Gamma_j^{\text{ph}}}\sigma_-^j a^\dagger)\Big] - L(\sqrt{\kappa} a) \tag{2}$$

where $L(x) = \frac{1}{2}\{x^\dagger x, \rho\} - x^\dagger \rho x$. γ_1 and γ_2 are the TLSs spontaneous emission rates, γ_1^d and γ_2^d the TLSs pure dephasing rates causing a Lorentzian broadening of their emission line FWHM given by γ_j^d. P is the TLSs incoherent pumping rate and κ the cavity mode loss rate. Γ_j^{ph} is the phonon scattering rate, describing the transfer of excitations from the TLS to the cavity mode. Γ_j^{ph} is a function of TLS-CM detuning and temperature, and was calculated using the following microscopic description of the exciton-phonon interaction[3, 4].
The transfer of excitations from the QD to the off-resonant CM via the absorption or emission of a phonon is described by the quantum collapse operator

$$L_j^{\text{ph}} = \sqrt{\Gamma_j^{\text{ph}}(\delta_j)}\sigma^- a^\dagger \tag{3}$$

that accounts for the decay from the state $|e\rangle \otimes |n=0\rangle$ to the state $|g\rangle \otimes |n=1\rangle$ at rate Γ^{ph}, with $|e\rangle$ ($|g\rangle$) the excited (ground) state of the exciton and $\{|n\rangle\}_{n\in\mathbf{N}}$ the Fock space of the quantized CM. Here we neglect the backscattering term $\sqrt{\Gamma_j^{\text{ph}}(-\delta_j)}\sigma^+ a$ describing the phonon-mediated feeding of the QD by the CM which is reasonable in the bad cavity regime ($\kappa \ll \gamma$)[3].
As shown by U. Hohenester using a Fermi golden rule approach[3], the phonon scattering rate Γ_j^{ph} accounting for the phonon-assisted decay of QD exciton into the CM is given by

$$\Gamma_j^{\text{ph}}(\delta_j) = \frac{2\pi}{\hbar^2}\left(\frac{g_0^j \cos(\theta)}{\delta_j}\right)^2 d(\delta_j) \tag{4}$$

with $d(\Omega) = \sum_{\boldsymbol{k}} |M_{\boldsymbol{k}}^e - M_{\boldsymbol{k}}^g|^2 [n(\omega_k)\delta(\Omega + \omega_k) + (n(\omega_k)+1)\delta(\Omega - \omega_k)]$ the effective phonon density of states[4]. $\boldsymbol{k}$ denotes the kth phonon mode of the phonon bath interacting with the QD exciton and $n(\omega) = \left[\exp(\frac{\hbar\omega}{k_B T} - 1)\right]^{-1}$ is the number of free phonons available at temperature T. $M_{\boldsymbol{k}}^\nu = \sqrt{\frac{\hbar k}{2\rho c_s V}} D_\nu \int d\boldsymbol{r} |\phi_\nu(\boldsymbol{r})|^2 e^{-i\boldsymbol{k}\cdot\boldsymbol{r}}$ is the exciton-phonon interaction matrix element[4], with c_s the speed of sound in the material, ρ the material's mass density, D_ν the deformation potential, V the phonon quantization volume and $\phi_\nu(\boldsymbol{r})$ the electronic wave function. An estimation of the effective phonon density of state can be computed by assuming the same Gaussian electronic wave functions for the exciton ground and excited states

$$\phi(\boldsymbol{r}) = \phi_g(\boldsymbol{r}) = \phi_e(\boldsymbol{r}) = \frac{1}{\sqrt{\pi^{\frac{3}{2}} \ell^2 \ell_z}} \exp\left(-\frac{\boldsymbol{u}^2}{2\ell^2} - \frac{z^2}{2\ell_z^2}\right) \tag{5}$$

where $\boldsymbol{u}$ denotes the QD growth plane directions and z the out-of-plane direction. Due to pyramidal QD growth characteristics, the effective confinement length ℓ_z along the growth direction is different from the one (ℓ) in the orthogonal plane. Using these Gaussian electronic wave functions, we computed the following effective phonon density of states

$$d(\Omega) = A\Omega^2(n(\Omega) - n(-\Omega) + 1) \exp\left(-\frac{\Omega^2\ell^2}{2c_s^2}\right) \frac{\operatorname{erf}\left(\frac{\Omega}{c_s}\sqrt{\frac{\ell_z^2-\ell^2}{2}}\right)}{\sqrt{\frac{(\ell_z^2-\ell^2)}{2}}} \tag{6}$$

with $A = \frac{\hbar D^2}{16\rho c_s^4 \pi^{\frac{3}{2}}}$ and $D = D_e - D_g$. This finally yields the following detuning dependent phonon scattering rate

$$\Gamma_j^{\mathrm{ph}}(\delta_j) = \frac{2\pi}{\hbar^2}\left(\frac{g_0^j\cos(\theta)}{\delta_j}\right)^2 A\delta_j^2(n(\delta_j) - n(-\delta_j) + 1) \exp\left(-\frac{\delta_j^2\ell^2}{2c_s^2}\right) \frac{\operatorname{erf}\left(\frac{\delta_j}{c_s}\sqrt{\frac{\ell_z^2-\ell^2}{2}}\right)}{\sqrt{\frac{(\ell_z^2-\ell^2)}{2}}}. \tag{7}$$

The master equation (2) is solved numerically using the quantum optics toolbox QuTip[5] to obtained the steady-state power spectra of the TLS and CM. The steady-state power spectrum is defined as

$$S(\omega) = \int_{-\infty}^{\infty} \lim_{t\to\infty} \langle A(t+\tau)B(t)\rangle\, e^{-i\omega\tau} d\tau \tag{8}$$

with $A, B = \sigma^+, \sigma^-, a^\dagger, a$. The correlation function $\langle A(t+\tau)B(t)\rangle$ is calculated numerically using the exponential-series based solver *essolve*[5] that computes the non-unitary time evolution of the system operators A and B by solving the master equation. The Fourier transform of the steady-state correlation function is then performed semi-analytically[5], giving the power spectrum, which is then used to compute a simulated spectrum directly comparable to the photoluminescence spectrum[1].

B. Dependence of emission intensity on detuning

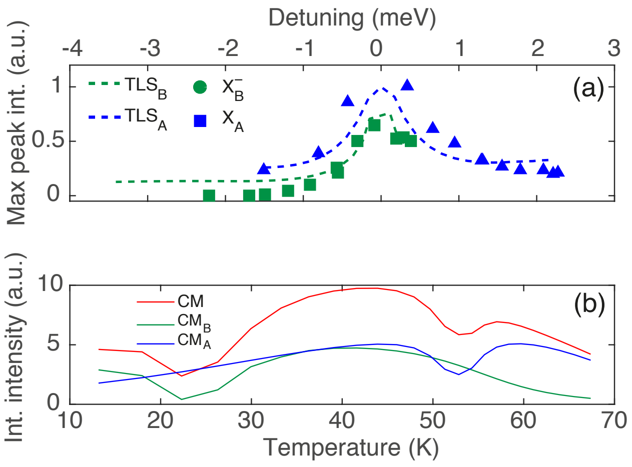

FIG. S 1. (a) Comparison of the maximum peaks intensities of the X_B^- and X_A transitions with the simulations. (b) Simulated CM integrated intensity as a function of temperature when both QDs are coupled to the cavity (red), compared to the same quantity when only QD A (blue) or QD B (green) is coupled to the cavity.

Fig. S1(a) compares the maximum peak intensity of the two QD features, extracted from the measured and simulated data using Lorentzian fits. The increase of the signal collected from both QDs, stemming from the Purcell reduction of their decay time near resonance, is in good agreement with the simulations. The best fit between the simulations and measured data was obtained using the same coupling strength g_0 for both QDs. This is consistent

with the position of the QDs within the L3 cavity that ensures both excitons couple equally with the mode. Although both QDs are equally coupled to the mode, the effective QD-cavity coupling strength is dependent on detuning. In particular, the off-resonant cavity emission varies with detuning. Having both QDs contributing equally to the CM off-resonant emission can thus be as important, in view of achieving superradiant or subradiant emission in QD-cavity systems, as for them to have equal QD-cavity coupling strength constants. To investigate this point, we compare in Fig. S1(b) the total off-resonant CM integrated intensity, to the contributions of each QD, labeled CM_A and CM_B. When the CM transition is in between X_B^- and X_A ($T = 40\,K$), both QD contribute equally to the cavity feeding. Moreover, this condition of equal cavity feeding is not very sensitive to temperature variations, and thus detuning, and remains valid for temperatures ranging from $30 - 50\,K$. The fact the off-resonant emission does not vary significantly with detuning is explained by the combined effect of pure dephasing and phonon scattering that greatly extend the detuning range of efficient cavity feeding.

C. Dependence on excitation power

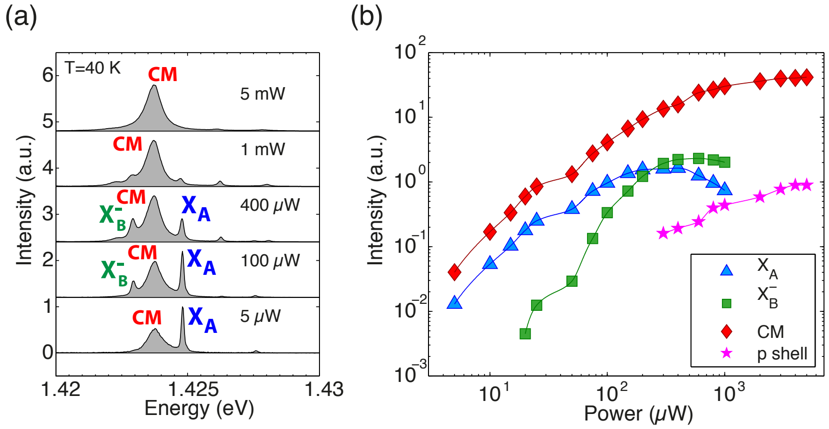

FIG. S 2. (a) Evolution of the PL spectrum of the coupled QD-cavity structure as a function of excitation power. The sample temperature is kept at $40\,K$, such that the cavity mode is spectrally centered between the two QDs (i.e. between X_A and X_B^-). (b) Analysis of the integrated intensities of the emission peaks as a function of excitation power.

Fig. S2 shows the excitation power dependence of the PhC cavity containing two weakly coupled QDs, as investigated in the present work (Figs. 3-5). What is striking about the observed spectral characteristics is that the CM does not saturate with the nearby s-shell states, namely X_B^- and X_A. This is very distinct from the behavior evidenced with single pyramidal QDs weakly coupled to a PhC cavity, for which we have previously reported that the power dependence of the CM mimics the linear increase as well as the saturation of the near-resonant QD exciton transition [6]. Another peculiar aspect seen in Fig. S2(a) is that at high excitation power ($P = 5$ mW) the QD peaks merge into a continuum dominated by the CM. Closer inspection of the integrated intensities in Fig. S2(b) reveals that when the excitation power is large enough to cause radiation from excited QD states of the p-shell, the trend of the CM begins to resemble the latter. As mentioned towards the end of the present article, we suspect that the particular features of the power dependence highlighted here may either hint towards the onset of cooperative emission, or to feeding from higher energy quantum wire states.

[1] C. Jarlov, E. Wodey, A. Lyasota, M. Calic, P. Gallo, B. Dwir, A. Rudra, and E. Kapon, Phys. Rev. Lett. **117**, 076801 (2016).
[2] M. Tavis and F. W. Cummings, Phys. Rev. **170**, 379 (1968).
[3] U. Hohenester, Phys. Rev. B **81** (2010).
[4] P. Kaer, T. R. Nielsen, P. Lodahl, A.-P. Jauho, and J. Mrk, Phys. Rev. B **86** (2012).
[5] J. R. Johansson, P. D. Nation, and F. Nori, Comput. Phys. Commun. **183**, 1760 (2012).

[6] M. Calic, P. Gallo, M. Felici, K. A. Atlasov, B. Dwir, A. Rudra, G. Biasiol, L. Sorba, G. Tarel, V. Savona, and E. Kapon, Phys. Rev. Lett. **106** (2011).
